# Nursing schools curricula: good hand hygiene guaranteed?

**DOI:** 10.1186/2047-2994-4-S1-P282

**Published:** 2015-06-16

**Authors:** DA De Wandel, S Blot, D Vogelaers

**Affiliations:** 1 Faculty of Education, Health & Social Work, University College Ghent, Belgium; 2 Faculty of Medicine and Health Sciences, Ghent University, Ghent, Belgium

## Introduction

Hand hygiene compliance is poor among graduated nurses. Hand hygiene behaviour is complex and shows great variation that can be attributed to the healthcare worker’s educational background.

## Objectives

The objective was to quantify the level of attention given to hand hygiene in the course curricula of nursing schools in Belgium.

## Methods

A questionnaire was distributed during the Federal Public Service’s (FPS) ‘hand hygiene campaign introduction session 2011’ for nursing school staff involved in teaching hand hygiene.

## Results

Response was 100% (n=49). The attending teachers represented 53% of all Belgian nursing schools. With each year of education, HH related knowledge (1st: 98%, 2nd: 49%, 3rd: 29%), attitude and skills (1st: 98%, 2nd: 71%, 3rd: 55%) receive less attention. Competencies are evaluated during clinical practice (90%), by means of written exams (90%), skills tests at school (37%) or ‘other’ (18%). Most popular teaching methods include live demonstration (98%), skills training sessions (71%) and video demonstration (59%). Courses are being held up to date using books (64%), publications of the Superior Health Council (44%), scientific publications (26%) and the FPS HH website (16%). Sixty-one per cent of the study books have not been updated in the past two years. The average time spent on infection control and hand hygiene was respectively 19.68hrs (sd 14.18, min 2 max 64) and 7.07hrs (sd 6.98, min 2 max 25).

## Conclusion

Hand hygiene education in Belgium shows great variation. Hand hygiene competencies are not systematically acquired nor assessed during the 3-year course. Moreover, teaching and assessment methods show considerable room for innovation.

## Disclosure of interest

None declared.

